# 3D-CT implanted interstitial brachytherapy for T2b nasopharyngeal carcinoma

**DOI:** 10.1186/1748-717X-5-113

**Published:** 2010-11-23

**Authors:** Yu-Feng Ren, Yuan-Hong Gao, Xin-Ping Cao, Wei-Jun Ye, Bin S Teh

**Affiliations:** 1 State Key Laboratory of Oncology in Southern China, Department of Radiation Oncology, Cancer Center, Sun Yat-sen University, 651 Dongfeng Road East, Guangzhou, PRC; 2 The Methodist Hospital, Department of Radiation Oncology, 6565 Fannin, DB1-077, Houston, Texas 77030, USA; 3 The Methodist Hospital Research Institute, 6565 Fannin, Houston, Texas 77030, USA

## Abstract

**Background:**

To compare the results of external beam radiotherapy in combination with 3D- computed tomography (CT)-implanted interstitial high dose rate brachytherapy (ERT/3D-HDR-BT) versus conventional external beam radiotherapy (ERT) for the treatment of stage T2b nasopharyngeal carcinoma (NPC).

**Methods:**

Forty NPC patients diagnosed with stage T2b NPC were treated with ERT/3D-HDR-BT under local anesthesia. These patients received a mean dose of 60 Gy, followed by 12-20 Gy administered by 3D-HDR-BT. Another 101 patients diagnosed with non-metastatic T2b NPC received a mean dose of 68 Gy by ERT alone during the same period.

**Results:**

Patients treated with ERT/3D-HDR-BT versus ERT alone exhibited an improvement in their 5-y local failure-free survival rate (97.5% vs. 80.2%, *P *= 0.012) and disease-free survival rate (92.5% vs. 73.3%, *P *= 0.014). Using multivariate analysis, administration of 3D-HDR-BT was found to be favorable for local control (*P *= 0.046) and was statistically significant for disease-free survival (*P *= 0.021). The incidence rate of acute and chronic complications between the two groups was also compared.

**Conclusions:**

It is possible that the treatment modality enhances local control due to improved conformal dose distributions and the escalated radiation dose applied.

## Introduction

In 1996, Chua *et al*. [1] reported the incidence of parapharyngeal extension in NPC patients to be as high as 72.5%. Of these patients, 65.5% exhibited extensions into the prestyloid space and/or the masticator space, which was associated with lower rates of 5-year local control and distant metastasis-free survival. Numerous studies also showed that parapharyngeal extension was an unfavorable prognosis factor in predicting overall relapse, local relapse, and distant metastasis [2-4].

Over the past few decades, two-dimensional brachytherapy (2D-BT) has been used to apply high doses of radiation directly to the primary tumor, while sparing nearby critical normal tissues. Some studies have reported that brachytherapy significantly enhances local control and the overall survival rate for early T-stage NPC [5,6], but 2D-BT may provide less adequate target volume coverage if the tumor has extended into the parapharyngeal space. 3D-computed tomography (CT)-implanted interstitial HDR brachytherapy (3D-HDR-BT) was developed from 2D-BT techniques [7-9], and with this approach, the target volume, as well as all of the vital structures involved, can be outlined using Plato software (PLATO BPS 14.2, Nucletron B.V, Veenendaal, The Netherlands). Reconstruction and 3D planning of interstitial implants with non-parallel applicators also use Plato software, as well as a real-time evaluation of the isodose distribution achieved in all CT slices. Accordingly, this interstitial implantation technique has been applied to the treatment of prostate cancer [10,11], breast cancer [12], rectal carcinoma [13], and cervical cancer [14]. However, there are no reports of the treatment results achieved using 3D-HDR-BT for the treatment of NPC.

Therefore, the goal of this current study is to evaluate the local control and distant metastasis events exhibited by stage T2b NPC patients that received ERT with or without additional 3D-HDR-BT therapy.

## Methods and Materials

### Patient characteristics

Forty NPC patients diagnosed with stage T2b disease at the Cancer Center of Sun Yat-Sen University (Guangzhou, People's Republic of China) between January 2004 and February 2008 were prescribed ERT with 3D-HDR-BT. To evaluate the efficacy of this treatment modality, these 40 cases were retrospectively compared with 101 patients diagnosed with non-metastatic T2b disease that were treated with ERT alone during the same period. For the two patient groups, the male/female ratio was about 3:1 with 104 males and 37 females, and the median age was 44 years (range, 19-80 years). Histological examination showed that 97.2% of the patients had World Health Organization (WHO) type II or III disease, 4 patients had type I disease.

Each patient was confirmed by biopsy, direct fiber-optic examination, complete blood count, blood chemistry test, chest X-ray, bone scan, and the extension of disease was further evaluated by physical examination, magnetic resonance imaging (MRI) of the nasopharynx and neck. Gross disease was carefully diagrammed for each patient and all MRI materials and clinical records were reviewed to minimize heterogeneity in restaging. Two radiologists specializing in head-and-neck cancers evaluated all scans and any disagreements were resolved by consensus. All stage T2b patients were restaged according to the American Joint Committee on Cancer staging system (6^th ^edition) [15]. Acute and late complications were scored according to criteria of the Radiation Therapy Oncology Group (RTOG) scoring system.

### Treatment methods

All patients were treated with definitive intent radiation therapy, and the external beam radiotherapy techniques have previously been reported [16]. After completion of primary radiotherapy, fiber optic nasopharyngoscopy was performed to assess tumor response. In the event of documented persistent or relapse disease, salvage treatments were provided, including brachytherapy, nasopharyngectomy or stereotactic radiotherapy (SRT).

For the ERT/3D-HDR-BT group, 40 patients received ERT followed by 3D-HDR-BT. Patients were immobilized in the supine position with a thermoplastic mask and treated with two lateral-opposing faciocervical portals to irradiate the nasopharynx and upper neck in one volume, followed by application of the shrinking-field technique to limit irradiation of the spinal cord. An anterior cervical field was used to treat the neck with a laryngeal block. The primary nasopharynx tumor received 60 Gy of ERT. Overall, the neck region received an accumulated radiation dose of 50 Gy, while the involved areas of the neck received 60-62 Gy. All patients were treated with one fraction daily for 5 days per week.

**Figure 1:**
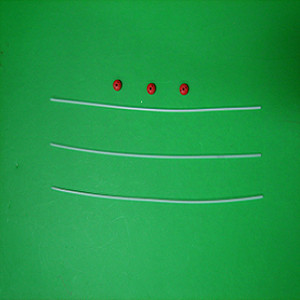
**A ProGuide Needle (189.601 ProGuide Needle Set 6F, sharp) was used as a nasopharyngeal applicator (microSelectron, Nucletron, nylon tube technique) for stage T2b NPC patients**.

**Figure 2:**
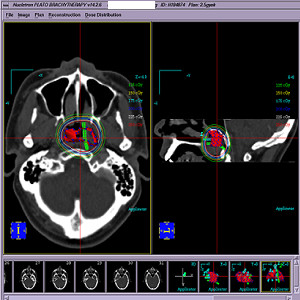
**Axial CT image of a NPC patient with stage T2b disease showing four ProGuide Sharp Needles in the parapharyngeal space with 2.5 GY, 2.25 GY, 2.0 GY, 1.75 GY, 1.50 GY, and 1.25 GY isodose lines**.

3D-CT-based interstitial brachytherapy was delivered using a high-dose-rate (HDR) afterloading machine (microSelectron, Nucletron, Veenendaal, The Netherlands), with ^192^-Ir as the source and ProGuide Needle (189.601 ProGuide Needle Set 6F, sharp) used as the nasopharyngeal applicator (microSelectron, Nucletron, nylon tube technique) (Figure 1). Patients were immobilized in the supine position with a thermoplastic mask and administered local anesthesia. The fiberoptic endoscope was guided to the treatment sites via the inferior meatus to the treatment positions. The interstitial portion of the implant consisted of inserting 2-4 stationary ProGuide Sharp Needles in the parapharyngeal tissues of the primary tumor site followed by immobilization of the applicators. The 3D treatment plan was performed as follows: 1) ProGuide Sharp Needles with an appropriate length for interstitial treatment were placed into the treatment volume, with the needles immobilized using the button sewed into the wings of the nose; 2) CT scanning using a 0.2 cm step to obtain 0.2 cm thick slices was performed after placement of the implants and CT images were subsequently transferred to a 3D treatment planning system (PLATO PBS 14.2); 3) the target volume (i.e. the nasopharyngeal primary tumor area and the parapharyngeal involved site) and ProGuide Sharp Needles were contoured; 4) non-parallel needles were reconstructed using the catheter reconstruction module of the PLATO BPS system before treatment plan was performed, so that any displacement of the needles could be identified and maintained within 1 mm;. 5) With all volumes defined, dwell positions were automatically activated within the target volume. Dose points were placed on the target surface and the prescribed reference dose was calculated from the mean dose achieved over all of the specified dose points on the target surface. Isodose surfaces, the dose homogeneity index, and coverage of the target volume by prescription dose were calculated and evaluated (Figure 2). All patients received 16GY over the entire implant volume.

Following treatment, applicator components were removed from the patients as follows: 1) applicators were disconnected from the indexer and flexible transfer tube; 2) needles were gently removed using standard interstitial techniques; 3) needles were discarded and the obturators were appropriately cleaned.

For the ERT alone group, 101 patients received radiotherapy delivered by a linear accelerator with 6 or 8 MV photons using the same ERT techniques. Primary nasopharynx tumors received a mean radiation dose of 68 Gy (range, 66-70 Gy), and all patients received 1 fraction daily for 5 days per week. During the period of ERT, all the patients were performed fiber optically every week to assess the tumor response to treatment.

Patients with AJCC-2002 N2-3 lymph nodal involved disease (19 in the ERT/3D-HDR-BT group and 43 in the ERT group) received neoadjuvant, concomitant, or adjuvant chemotherapy (all cisplatin-based).

### Statistical analysis

All survival rates were calculated from the date of histological diagnosis using SPSS version 13.0 (SPSS, Inc., Chicago, IL). The following endpoints (interval to the first defining event) were assessed: local failure-free survival (LFFS), nodal failure-free survival (NFFS), overall survival (OS), disease-free survival (DFS), distant metastasis-free survival (DMFS), and disease-specific survival (DSS). The chi-square test was used to compare clinical-pathological characteristics between the two groups. Estimates of actuarial survival rates were based on the method of Kaplan-Meier [17]. The log-rank test was used to compare survival curves [18], while multivariate analyses were performed using the Cox proportional hazards model [19]. A *P*-value ≤ 0.05 was considered statistically significant.

## Methods

Forty NPC patients with stage T2b NPC were treated with ERT/3D-HDR-BT under local anesthesia. These patients received 60 Gy of ERT, followed by 16 Gy administered by 3D-HDR-BT. Another 101 patients received a mean dose of 68 Gy by ERT alone during the same period. The median age, gender, AJCC stage, N-category, histological grade, and chemotherapy treatment regimen for the ERT/3D-HDR-BT group and the ERT group were not significantly different (Table 1).

**Table 1:** Characteristics of 141 NPC patients at presentation

Patient Characteristics	ERT/3D-HDR-BT n (%)	ERT n (%)	*P*-value
**Age (years)**			0.743
≤ 45	23 (57.5%)	55 (54.5%)	
> 45	17 (42.5%)	46 (45.5%)	
**Gender**			0.252
Male	34 (85.0%)	77 (76.2%)	
Female	6 (15.0%)	24 (23.8%)	
**Histology**			0.956
WHO type I	1 (2.5%)	3 (3.00%)	
WHO type II/III	39 (97.5%)	98 (97.0%)	
**UICC stage**			0.064
II	32 (80.0%)	89 (88.1%)	
III	6 (15.0%)	12 (11.9%)	
IV a	2 (5.0%)	0 (0%)	
**N stage**			0.282
N0-1	21 (52.5%)	58 (57.4%)	
N2	17 (42.5%)	43 (42.6%)	
N3	2 (5.0%)	0	
**Therapy**			0.264
Received	19 (78.4%)	43 (81.4%)	
Not received	21 (14.9%)	58 (1.5%)	

WHO: World Health Organization; UICC: International Union Against Cancer.

### Local and regional control

**Figure 3:**
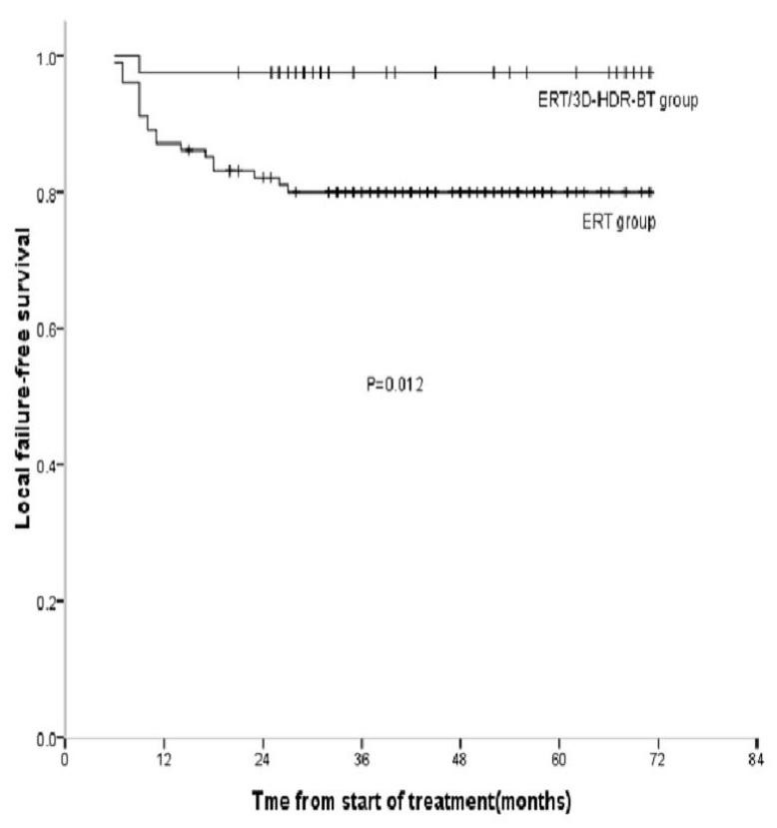
**Five-year actuarial local failure-free survival (LFFS) curves for ERT/3D-HDR-BT vs. ERT groups**.

One patient in the combined treatment group and 20 patients in the ERT group developed a local recurrence. The 5-year actuarial LFFS rates for the ERT/3D-HDR-BT group and the ERT group were 97.5% and 80.2%, respectively (*P *= 0.012). For local control, the additional 3D-HDR-BT therapy received by patients was found to be significant according to the log-rank test (Figure 3). Two patients in the ERT/3D-HDR-BT group and 1 patient in the ERT group experienced recurrence of neck nodal, and the 5-year actuarial NFFS rate for each of the treatment groups was 99.0% and 95.0%, respectively (*P *= 0.141). Furthermore, the local regional survival rate was 92.5% and 79.2%, respectively, for the two treatment groups (*P *= 0.066).

### Survival rates

**Figure 4:**
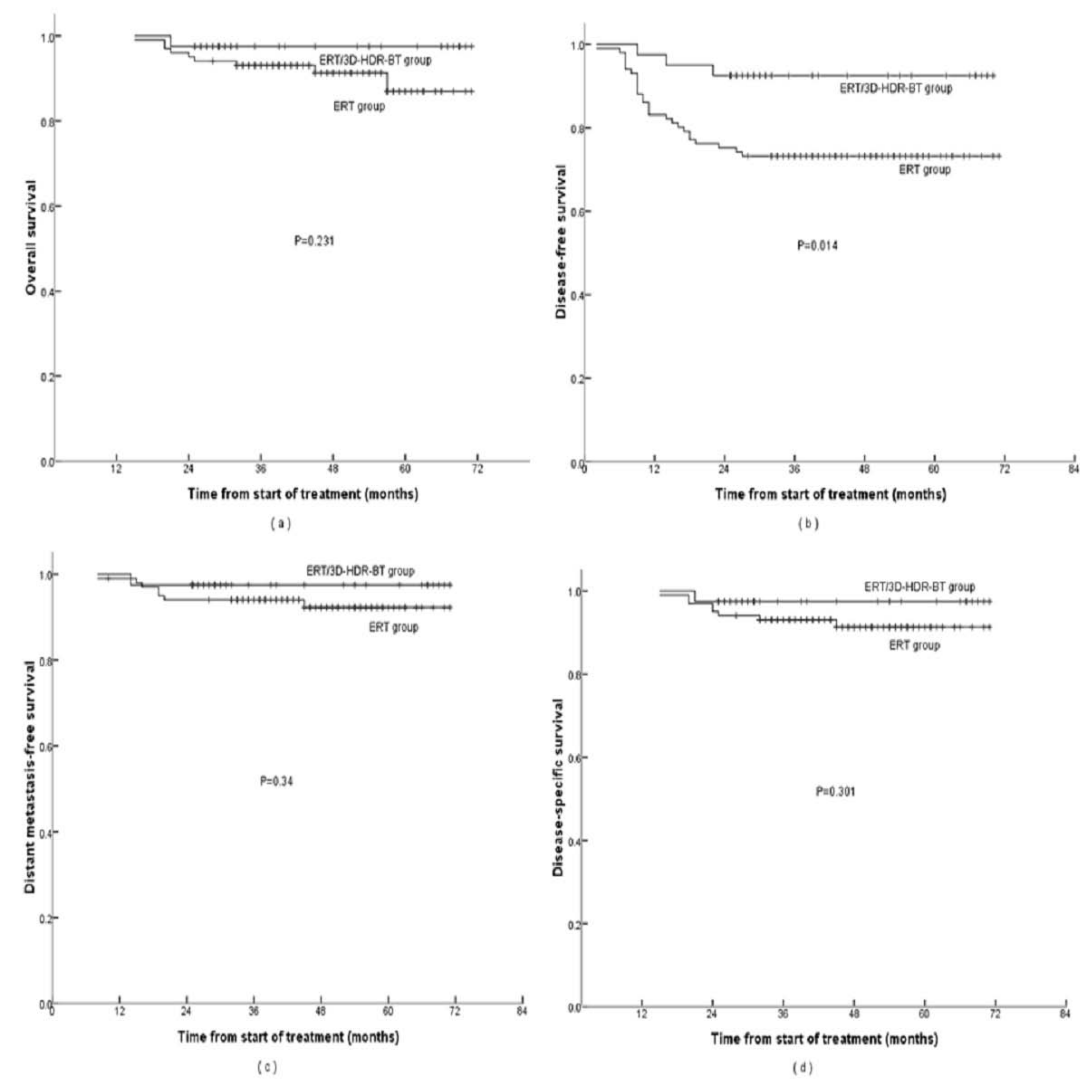
**Five-year actuarial survival curves for patients treated with ERT/3D-HDR-BT vs. ERT: (a) overall survival (OS); (b) disease-free survival (DFS); (c) distant metastasis-free survival (DMFS); and disease-specific survival (DSS)**.

The 5-year actuarial OS, DFS, DMFS, and DSS curves for the ERT/3D-HDR-BT group versus the ERT group are shown in **Figure **4 a-d. The specific survival rates were 97.5% and 91.1% (*P *= 0.231), 92.5% and 73.3% (*P *= 0.014), 97.5% and 92.1% (*P *= 0.301), and 97.5% and 93.1% (*P *= 0.340), respectively. The difference in DFS rate was significantly different for the two groups. There was no trend of improvement observed for the other endpoints between the two groups.

### Multivariate analyses

Potential prognostic factors, including gender, age (≤ 45 y vs. > 45 y), N-stage (0-3), and treatment factor (administration of 3D-HDR-BT), were included in a Cox proportional hazards model with backward elimination of the insignificant explanatory variables. The results of the multivariate analysis were summarized in Table 2, and administration of 3D-HDR-BT was identified as a favorable prognostic factor for LFFS, the ERT/3D-HDR-BT group was found to have lower hazard rates of LFFS than the ERT group (Cox regression: *P *= 0.046, RR = 0.129, 95% CI: 0.017 - 0.968). When all of the potential prognostic factors associated with 3D-HDR-BT were fitted into the Cox model, the parameter estimate of the administration of 3D-HDR-BT changed by only 2%, and 3D-HDR-BT remained the only significant prognostic factor for determining the ultimate LFFS rate. The Cox proportional hazard analysis also showed that administration of 3D-HDR-BT was a favorable prognostic factor for DFS (Cox regression: *P *= 0.021, RR = 0.181, 95% CI: 0.042-0.774). In contrast, N-category was found to be an unfavorable prognostic variable for the endpoints of OS, DMFS, and DSS.

**Table 2:** Summary of multivariate analysis of prognostic factors

Endpoint	Factors	B	*P*-value	Exp(B)	95% CI
**LFFS**	UICC stage	NS			
	N stage	NS			
	3D-HDR-BT: no vs. yes	-2.048	0.046	0.129	0.017-0.968
	Chemotherapy	NS			
**NFFS**	UICC stage	NS			
	N stage	NS			
	3D-BT: no vs. yes	NS			
	Chemotherapy	NS			
**OS**	UICC stage	7.979	0.005	4.729	1.609-13.898
	N stage	NS			
	3D-HDR-BT: no vs. yes	NS			
	Chemotherapy	NS			
**DFS**	UICC stage	NS			
	N stage	NS			
	3D-HDR-BT: no vs. yes	-1.709	0.021	0.181	0.042-0.774
	Chemotherapy	NS			
**DSS**	UICC stage	NS			
	N stage	1.428	0.016	4.168	1.307-13.299
	3D-HDR-BT: no vs. yes	NS			
	Chemotherapy	NS			
**DMFS**	UICC stage	NS			
	N stage	1.436	0.015	4.205	1.322-13.378
	3D-HDR-BT: no vs. yes	NS			
	Chemotherapy	NS			

UICC: International Union Against Cancer; NS: not significant; CI: confidence interval.

### Complications

All 40 patients in the ERT/3D-HDR-BT group experienced small amounts of blood loss, which was successfully managed with conservative treatment measures so that none of the patients required blood-transfusions during the course of treatment. Overall, treatment-related complications were generally controlled within the two different treatment groups. Of the patients with N-stage (N2-3) disease in the two groups that were treated with concurrent chemo-radiotherapy (DDP 40 mg/m^2 ^per week), 9 patients experienced Radiation Therapy Oncology Group (RTOG) grade III-IV hematological toxicity, 11 patients experienced grade III mucositis, and no cases of severe epistaxis required blood transfusions.

The most common radiation-related complication was xerostomia. Various degrees of xerostomia were seen commonly, and 37 patients developed xerostomia during their course of radiotherapy, 8 of which were from the ERT/3D-HDR-BT group. Other side effects experienced included different grades of hearing impairment (n = 35), with 5 patients experiencing grade 1/2 hearing impairment in the ERT/3D-HDR-BT group, and 1 patient undergoing a complete loss of hearing (i.e. grade 4) in the ERT group. One patient in the ERT group developed a limited ability to open their mouth (2.5 cm), and incidences of pituitary-endocrine dysfunction, neck fibrosis limiting movement, temporal lobe necrosis, and cranial nerve palsy were not seen for patients in the two groups. In addition, no significant late toxicities directly due to the implantation interstitial technique were seen. Complications of nasopharyngeal middle ulceration/necrosis were identified for 7 patients in the ERT/3D-HDR-BT group exhibiting foul-smelling crust, and for 19 patients in the ERT group exhibiting light to middle ulceration/necrosis.

## Discussion

The role of routine intracavity brachytherapy for T2b NPC patients at initial diagnosis remains controversial [6,20-23]. Some oncologists consider the dose distribution achieved with routine brachytherapy to be far from optimal when the parapharyngeal space is involved, especially for cases of advanced parapharyngeal invasion [24]. In 2005, Leung *et al*. [25] reported excellent local control for 34 NPC patients with T2b disease that received routine brachytherapy. In addition, there have been many improvements in imaging technology and treatment techniques, particularly with the development of 3D-HDR-BT from 2D-BT. In this study, we retrospectively analyzed the treatment results for stage T2b NPC patients that received ERT/3D-HDR-BT versus ERT alone.

In a comparison of the treatment results for the two patient groups, a 17.3% absolute improvement in local control rate was identified for the 3D-HDR-BT group. This contributed to an overall improvement in the 5-year LFFS rate for the ERT/3D-HDR-BT group that was also accompanied by an acceptable level of acute and late treatment-related toxicity. We hypothesized that this rate of improvement was due to more efficient conformal dose distributions and the escalation of radiation dose that was able to be achieved with ERT/3D-HDR-BT. This hypothesis was supported by evidence of a dose-response relationship for NPC that was found to be greater than the conventional tumoricidal level previously achieved [26-28]. These results were consistent with other studies, where ERT/3D-HDR-BT had been shown to gain excellent local control and increase survival rates for patients with prostate cancer [10,11], breast cancer [12], locally advanced rectal carcinoma [13], and cervical cancer [14]. Given the rapid fall-off in radiation that is characteristic of treatment with ^192-^Ir, 3D-HDR-BT provides the opportunity to increase the radiation dose directly administered to the nasopharyngeal mucosa and the parapharyngeal space.

In this study, one patient in the 3D-HDR-BT group developed local recurrence. Given that the dose distribution of 3D-HDR-BT is able to cover the target volume for the patients with parapharyngeal space involvement, and the observation that tumor recurrence is within the primary gross tumor site, the possibility that a region of tumor will not receive radiation is little with this technique. Therefore, we hypothesize that local failures that do occur despite these considerations, are a result of intrinsic radioresistance of the tumor. In this study, the ERT/3D-HDR-BT combined treatment modality achieved a high probability of local control while limiting radiation dose to normal structures, thereby demonstrating that patients with non-metastatic stage T2b NPC can be effectively treated with the 3D-HDR-BT implantation interstitial technique. Furthermore, these results contribute to our understanding of the benefits of treating T2b tumors with 3D-HDR-BT.

Compared with the ERT group, an improvement in DFS was achieved with 3D-HDR-BT, and this was statistically significant for stage T2b patients. According to multivariate analyses, administration of 3D-HDR-BT was associated with a significantly lower disease failure rate. Therefore, since the administration of 3D-HDR-BT did not reduce either nodal or distant failure rates, the improvement in the 5-year DFS is hypothesized to be the result of a reduction in local failure.

Two patients in the ERT/3D-HDR-BT group and one patient in the ERT group experienced neck nodal recurrences, indicating that treatment with 3D-HDR-BT did not improve regional nodal control rates. However, both groups achieved excellent regional control due to the biological characteristic of metastasis neck nodes which have been reported to be radiocurable [29]. Previously, Yeh *et al*. [20] reported that elective irradiation of all cervical lymphatics should be performed. However, while all node-negative patients received at least 46.8 Gy of elective neck irradiation, only 1.2% developed regional relapse.

Compared with the ERT group, patients of the ERT/3D-HDR-BT group did not show improvements in their rates of OS, DMFS, and DSS. All patients with N2-3 category tumors received chemotherapy-radiotherapy of DDP at 40 mg/m^2 ^per week, and benefits were associated with the administration of chemotherapy for locally advanced NPC. In a previous study, adjunct chemotherapy was found to further provide significantly higher local regional control and survival rates [30], while other studies showed an overall survival benefit was achieved with the administration of concurrent and adjuvant chemotherapy [31,32]. In this study, no improvement in patient survival was observed for either treatment group, although the relatively short patient follow-up interval may have had a role in this observation. Further studies will be needed with longer follow-up periods in order to confirm the treatment results of this study.

The interstitial implantation procedure was found to be well tolerated by all of the T2b patients that received ERT/3D-HDR-BT. While some patients did experience small amounts of blood loss over the course of the interstitial implantation procedure, epistaxis was managed with conservative treatment measures and was always self-limiting. None of the patients required blood-transfusions due to excessive bleeding during the course of treatment.

In the ERT group, one patient presented with limited mouth opening, and this case emphasizes the importance of a thorough anamnesis and examination in order to discover such phenomena as early as possible. For cases with this condition, treatment should be restricted to palliative dental treatment, excluding any surgical procedure.

The most common radiation-related complication experienced was xerostomia. The incidence rate of xerostomia was found to differ significantly between the two treatments groups, probably due to the rapid dose falloff associated with ERT/3D-HDR-BT that could achieve a high probability of local control while limiting the exposure of normal vital tissues to the radiation dose. Furthermore, patients treated with ERT/3D-HDR-BT did not report any major complications or any significant late toxicity directly due to the implantation procedure.

Despite the improvement in patient treatment that can be also achieved using 3D techniques, the availability of this technology is limited in many countries. However, the accessibility of afterloading machines, coupled with a relatively simple procedure, makes brachytherapy an attractive technique for centers that do not have three-dimensional facilities, or that have tight machine times, as is often the case in developing countries. Novel radiotherapy treatment equipment used for intensity-modulated radiation therapy (IMRT), image-guided radiation therapy (IGRT), and three-dimensional conformal radiation therapy (3D-CRT), all have been shown to achieve excellent local control and survival outcomes in the treatment of patients with early stage T NPC. However, the cost of these novel technologies is very high [33-35]. When treatment costs were analyzed at our institution, ERT/3D-HDR-BT was found to reduce personnel cost while still provide good treatment results.

Our study was limited by the non-randomized treatment performed due to the retrospective approach of our study. However, disease characteristics and parameters were comparable between the two groups analyzed, and attempts were made to reduce any potential bias present in this study in order to achieve more homogeneous patient groups.

## Conclusions

The application of an interstitial implantation brachytherapy technique that uses 3D treatment plan system (PLATO BPS 14.2) was evaluated for the treatment of stage T2b NPC patients. Although the technique is relatively complicated, it can be performed with well tolerated complications. Overall, the administration of interstitial 3D-HDR-BT achieved excellent local control for stage T2b NPC patients as a result of improved target coverage and conformality of the radiation dose applied. However, additional prospective, randomized studies are needed to confirm the results of this study.

## Competing interests

The authors declare that they have no competing interests.

## Authors' contributions

YFR - Primary author of manuscript and revisions. YHG - Contributed to writing of manuscript and concept. WJY - Performed physics plans and assisted with manuscript. XPC - Concept of paper, contributed in writing manuscript and all revisions, BST- Concept of paper, contributed in writing manuscript and all revisions. All authors read and approved the final manuscript.
